# Through the Looking Glass: Updated Insights on Ovarian Cancer Diagnostics

**DOI:** 10.3390/diagnostics13040713

**Published:** 2023-02-14

**Authors:** Sourav Chakraborty, Priti S. Shenoy, Megha Mehrotra, Pratham Phadte, Prerna Singh, Bharat Rekhi, Pritha Ray

**Affiliations:** 1 Imaging Cell Signaling and Therapeutics Lab, Advanced Centre for Training Research and Education in Cancer, Navi Mumbai 410210, India; 2 Homi Bhabha National Institute, BARC Training School Complex, Anushaktinagar, Mumbai 400094, India; 3 Tata Memorial Hospital, Dr. E Borges Road, Parel, Mumbai 400012, India

**Keywords:** epithelial ovarian cancer, diagnosis, biomarkers, prognosis

## Abstract

Epithelial ovarian cancer (EOC) is the deadliest gynaecological malignancy and the eighth most prevalent cancer in women, with an abysmal mortality rate of two million worldwide. The existence of multiple overlapping symptoms with other gastrointestinal, genitourinary, and gynaecological maladies often leads to late-stage diagnosis and extensive extra-ovarian metastasis. Due to the absence of any clear early-stage symptoms, current tools only aid in the diagnosis of advanced-stage patients, wherein the 5-year survival plummets further to less than 30%. Therefore, there is a dire need for the identification of novel approaches that not only allow early diagnosis of the disease but also have a greater prognostic value. Toward this, biomarkers provide a gamut of powerful and dynamic tools to allow the identification of a spectrum of different malignancies. Both serum cancer antigen 125 (CA-125) and human epididymis 4 (HE4) are currently being used in clinics not only for EOC but also peritoneal and GI tract cancers. Screening of multiple biomarkers is gradually emerging as a beneficial strategy for early-stage diagnosis, proving instrumental in administration of first-line chemotherapy. These novel biomarkers seem to exhibit an enhanced potential as a diagnostic tool. This review summarizes existing knowledge of the ever-growing field of biomarker identification along with potential future ones, especially for ovarian cancer.

## 1. Introduction

Despite the incredible advances in the field of medical science, epithelial ovarian cancer (EOC) remains a challenge that affects the population globally. The most lethal of all gynaecological malignancies, EOC accounts for a sizable amount of all cancer deaths in women worldwide [1]. EOC is also called the “silent lady killer” because of its relatively asymptomatic nature during the early course of the disease. Almost 70% of patients that are diagnosed with EOC present with clinically advanced stage of disease (stage III and IV) [2], highlighting the need for more accurate and reliable biomarkers for EOC.

Biomarkers can be classified into three types: predictive, diagnostic, and prognostic [3]. A predictive biomarker serves as a tool to predict clinical outcome. These help in optimizing specific treatment for patients and can predict their response to treatment. Diagnostic biomarkers serve a role in a more accurate diagnosis. These can be stage- and subtype-specific. Prognostic biomarkers provide information about the overall outcome of the patient, including the expected development of the disease, irrespective of any treatment or therapeutic intervention.

An ideal biomarker is one which can be used in an extensive screening process, enabling clinicians to diagnose asymptomatic patients. Detection of an ideal biomarker must be inexpensive as well as non-invasive. A substance secreted exclusively by tumour tissues but not normal tissues or tumour-specific antigens that can be detected in body fluids are examples of an ideal biomarker. The performance as well as the clinical suitability of a biomarker is further evaluated by its sensitivity and specificity. Sensitivity is the ability to detect a disease in patients wherein the disease is truly present (i.e., a true positive) and specificity is the ability to rule out the disease in patients where the disease is truly absent (i.e., a true negative) [4].

Currently, only a few biomarkers are used clinically, owing to their high sensitivity and specificity for the detection of EOC [5]. Strategies for early-stage detection of ovarian cancer must have high sensitivity (>75%) as well as a high specificity (99.6%) to attain a positive predictive value of at least 10%, considering the low prevalence of the disease [6]. Such high specificity currently cannot be attained with the existing screening methods and will likely not be attained by adopting a singular testing strategy. Thus, the discovery of novel ovarian cancer-specific molecular biomarkers/panels are emerging as important avenues toward early detection.

## 2. Currently Used Biomarkers in Epithelial Ovarian Cancer

### 2.1. Serum-Based Biomarkers

#### 2.1.1. Cancer Antigen 125 (CA-125)

CA-125, also known as Mucin 16 (MUC16), is a glycosylated 200 kDa transmembrane mucin protein encoded by the MUC16 gene. It is expressed on the surface of müllerian-origin and mesothelial cells and is released from the cell surface by proteolytic cleavage [7]. Bast et al. in 1981 discovered CA-125, which has emerged as a widely used tumour marker for EOC with a serum threshold of 35 U/mL [8]. Compared to other serum biomarkers (CA-19-9, CEA), CA-125 has shown better diagnostic performance and can distinguish between benign and EOC tumours [9]. Subsequently, the Food and Drug Administration (FDA) approved the use of CA-125 for cancer surveillance in women diagnosed with EOC [5]. About 85% of EOC patients have elevated levels of CA-125; however, high serum levels of CA-125 can also be indicative of other gynaecological diseases such as endometriosis, liver cirrhosis, uterine fibroids, gastrointestinal cancer, etc., thereby limiting its specificity and sensitivity to some extent [10]. Therefore, for reliable early detection methods, complementary biomarkers are needed. In the European Prospective Investigation into Cancer and Nutrition (EPIC) cohort study, anti-CA-125 autoantibodies in combination with CA-125 antigen was observed to be a better early detection biomarker strategy compared to the use of individual ones [11].

#### 2.1.2. Human Epididymis Protein 4 (HE4)

HE4 protein, originally isolated from human epididymis, is a 25 kDa protein expressed in the reproductive and respiratory tracts [12]. HE4 is secreted extracellularly as glycoprotein into the bloodstream and detected at high levels in serous and endometroid EOC patients [13,14]. In a study by Anastasi et al., 31 of 32 EOC patients (96.9%) had HE4 in their serum [15]. Subsequent analysis conducted by Scatella et al. revealed that HE4 levels were present 5–6 months before CA-125 levels were discovered [14]. A meta-analysis conducted by Yu et al. revealed a pooled sensitivity and specificity of 80% and 91.6%, respectively, for HE4 compared to 66% and 87.1% for CA-125 [16]. Combination of HE4 and CA-125 was proven to be a more robust early diagnostic marker strategy than CA-125 alone [11,12,13]. Further prospective study found that the combination of CA-125, HE4, and computed tomography (CT) is more effective at predicting the presence of residual tumours post-NACT [17]. Additionally, HE4 received FDA approval for its preoperative use in recurrence assessment and early detection in EOC patients.

To improve the efficacy of prediction and/or diagnosis, multi-gene panel markers are often utilized, which results in higher accuracy and in overcoming heterogeneity.

### 2.2. Multivariate Index Assays for Epithelial Ovarian Cancer

#### 2.2.1. Ovarian Malignancy Algorithm (OVA1)

OVA1 is a multivariate index assay based on five proteins and is the first FDA-approved (2009) preoperative serum biomarker test for EOC. It combines serum levels of CA-125 with four other inflammatory and transport proteins: microglobulin beta2, transthyretin, ApoA1, and transferrin [10]. The calculation of the OVA1 algorithm is based on serum levels of these five proteins in combination with imaging data and the menopausal status of ovarian cancer patients. This algorithm can distinguish malignant masses from benign pelvic masses and achieves a sensitivity of 96% with 28% specificity in postmenopausal women and 85% with 40% specificity in premenopausal women [18].

#### 2.2.2. Risk of Ovarian Malignancy Algorithm (ROMA)

ROMA, developed by Moore et al., includes the patient’s menopausal status along with two biomarkers, HE4 and CA-125, to diagnose ovarian cancer [19]. This algorithm was observed to achieve a sensitivity of 93%, a specificity of 75%, a negative predictive value of 93–94%, and an accuracy of 83% in diagnosing early-stage disease. Huy et al. showed that ROMA provided better prediction of preoperative recurrence than CA-125 and HE4 alone [20]. A successful prospective, multi-centre, blinded clinical trial by Moore et al. showed ROMA achieved a sensitivity of 92.3% and a specificity of 76.0% for postmenopausal women and a sensitivity of 100% and specificity of 74.2% for premenopausal women suffering from EOC [21]. Based on the results of clinical trials, ROMA received FDA approval in the US for differentiating malignant ovarian cancer and benign pelvic masses.

#### 2.2.3. Risk of Ovarian Cancer Algorithm (Overa)

In 2016, the FDA approved a new, modified version of OVA1 that came out as the second-generation OVA1 test, originally called OVA2 but now trademarked as Overa [22]. This algorithm replaces two proteins from OVA1 (transthyretin and beta-2 microglobulin) with HE4 and follicle-stimulating hormone (FSH). Overa not only allows the identification of EOC but also indicates the severity of the condition and the course of treatment. Overa was found to have high diagnostic sensitivity and improved specificity compared to other methods.

Taken together, the combination of these assessment methods seems beneficial for overall diagnosis and prognosis and in determining the risk of malignancy. However, specific biomarkers are also required to distinguish between the subtypes of EOC, which are genetically and histologically heterogeneous.

### 2.3. Genomic Profiling in Epithelial Ovarian Cancer

#### Germline/Somatic BRCA1/2 Mutations and HRD Status

Whole-genome analysis of EOC has led to the identification of certain genes that may cause susceptibility to ovarian cancer and may be used as a predictor of ovarian cancer development. Genomic profiling of ovarian cancer identifies genes that can not only predict susceptibility to ovarian cancer but also help distinguish between various ovarian cancer subtypes [23]. DNA repair plays an important role in the efficacious repair of genomic insults in order to maintain normal cellular functions and prevent genome-wide instability [24]. Therefore, mutations in DNA repair genes increase the susceptibility of the patient to developing cancer. A plethora of genes have been implicated in the DNA damage response pathway and are further grouped into five distinct pathways depending on their function [25]. Out of the different types of damage incurred by the DNA, double-strand breaks (DSB) are considered to be the most harmful genetic insult and if left unrepaired can cause severe genomic instability leading to cell death [26]. The homologous recombination (HR) pathway is one of the two pathways deployed in the cell to take care of these DSBs [25]. The HR pathway is thought to provide high-fidelity and template-dependent repair of these DNA damages [27]. Impairment/mutations in the HRR genes have invariably been associated with an increased susceptibility to multiple forms of cancer. However, they are predominantly observed in breast and ovarian cancer and are called homologous recombination deficiency (HRD) tumours [28]. The analysis conducted by TCGA showed that mutations in BRCA1/2 play a crucial role in HGSOC development. Germline mutations in BRCA1/2 were observed in 8% and 9% of the patients respectively [29]. Furthermore, women harbouring these hereditary mutations are at a higher risk of developing breast cancer (60–80%) and ovarian cancer (20–40%), classified as hereditary breast and ovarian cancer (HBOC) [30]. In a clinical setting, the HRD phenotype in patients is adjudged upon evaluating their HRD score. These patients are treated using Poly (ADP-ribose) polymerase (PARP) inhibitors (PARPi), as such tumours predominantly rely on the base excision repair mechanism to repair subsequent genetic insults [31]. PARP is critical in DNA damage response, as it plays a central role in both nucleotide excision repair (NER) and base excision repair (BER) [32]. It is responsible for sensing single-strand breaks (SSBs) and is recruited there to further relay the repair signalling [33]. PARPis work by catalytically inhibiting/trapping PARP, which leads to the generation of massive double-stranded breaks, causing cell death via synthetic lethality [34]. Sims et al. conducted a retrospective study to determine the correlation between HRD status and the clinical outcome of patients with advanced ovarian cancer. They observed that patients with germline or somatic BRCA mutations had much better overall survival (OS) and progression-free survival (PFS) in comparison to the HRD-negative cohort in regard to PARP inhibitors (PARPi), indicating that HRD status has a prognostic value in ovarian cancer [35]. Another retrospective study of the SCOTROC4 clinical trial by Stronach et al. revealed that HRD patients showed improved PFS and OS compared to HRD-negative patients in regard to escalation of platinum dosage [36]. Results from different prospective clinical trials also indicate that HRD status can be used as a biomarker for PARPi therapy response [37]. However, there is a lack of uniformity in the HRD test that is used in clinics for assessing HRD status. These HRD tests belong to three main categories. The first includes identification of loss-of-function mutations in key HRR genes such as BRCA1, BRCA2, RAD51C, RAD51D, and PALB2. The second involves evaluating the patterns of allelic imbalances such as LOH and copy-number variations in the HRR genes. The third involves functional assays for the real-time determination of HRD [38]. Therefore, it is important to integrate these assays to create robust and composite markers to devise an effective therapeutic regimen for treating HRD-positive ovarian tumours.

### 2.4. Immunohistochemistry-Based Biomarkers for Subtype-Specific Diagnosis and Prognosis of EOC

#### 2.4.1. Serous Carcinomas

Serous EOC is the most common and accounts for approximately 80% of epithelial carcinomas. It usually presents as large bilateral tumours and exhibits a mixture of cystic, papillary, and solid growth patterns [39]. Serous ovarian carcinomas comprise two distinct types i.e., high-grade serous (HGSOC) and low-grade serous (LGSOC), which originate from separate precursor lesions and show distinct molecular profiles and clinical presentations [40]. The HGSOC subtype accounts for approximately 85–90% of serous carcinomas and 70% of cases of EOC. One of the most well-established theories regarding the origin of HGSOCs suggest that they originate from müllerian epithelial precursor lesions that are present on the fallopian tube and not from the ovary. This theory is based on the observation that women carrying germline mutations in BRCA1/2 show a presence of dysplastic epithelium in the fallopian tube. Serous tubal epithelial carcinoma (STIC) is believed to be the immediate precursor of HGSOC in the fallopian tube. STIC cells can detach from the fallopian tube and disseminate into the ovary, which gives rise to HGSOCs [41]. HGSOCs are generally detected at an advanced stage, leading to a poor prognosis [42,43]. As compared to HGSOC, the LGSOC subtype is infrequent, accounting for 10–15% of cases of serous carcinomas and <5% of EOC cases; it has an intermediate prognosis [44]. LGSOC is considered to arise in a stepwise manner from serous borderline tumours, which could either be invasive or non-invasive. These are diagnosed at a younger age and are largely unresponsive to standard chemotherapy regimens [45]. Various protein-based biomarkers for distinguishing serous carcinomas from other EOCs and HGSOC from LGSOC are discussed below.

##### Diagnostic Biomarkers

WT-1 (Wilms tumour 1): *WT-1* was first discovered as a tumour suppressor gene and is now known to have a role as a transcription factor. It is known to play a role in mRNA metabolism and is overexpressed in many adult cancers such as breast, colorectal, etc. Taube et al. have demonstrated the utility of WT-1 as an independent prognostic and diagnostic marker in a cohort of primary high-grade serous carcinoma patients (n = 207) [46]. Currently, WT-1 is routinely utilised as an independent diagnostic marker of HGSOC to distinguish HGSOC from other subtypes. WT-1 might be uncommonly positive in endometrioid carcinoma, but almost never in clear cell carcinoma [47,48,49].

P53: Although a large number of genes play a role in tumorigenesis, *p53* is one of the key tumour suppressors, regulating various signalling pathways. P53 plays a role in cell cycle, DNA repair, senescence and is also known to transcriptionally regulate an array of genes involved in apoptosis, autophagy, etc. [50]. *p53* mutations occur in almost all cancer types and range from approximately 10% (hematopoietic malignancies) to nearly 95% in HGSOC; they can be seen as diffuse and strong nuclear expression or in the form of a total loss of expression by immunohistochemistry (IHC) [51]. Mutations in tumor suppressor p53 are rarely present in LGSOC, which can be seen in the form of focal expression by IHC.

P16INK4A: *p16Ink4a* is a tumour-suppressor gene and an important cell cycle regulator during the transition from G1 to S phase. Approximately 60–80% of HGSOC patients show immunoreactivity for p16Ink4a, which can be seen as diffuse staining by IHC [52].

Sallum et al. have described a p53/p16 index to distinguish between HGSOC and LGSOC tumours, which has been summarized in Table 1 [52].

##### Prognostic Biomarkers

Research conducted by Sehouli et al. highlights that LGSOC patients expressing high Ki-67 levels show a significant decrease in OS (34 months vs. 46 months) and a drastic reduction in 5-year PFS [53]. Furthermore, in a cohort of 55 LGSOC patients, Fernandez et al. reported that high expression levels of oestrogen receptors were associated with better survival outcome [54]. However, these studies require further validation in a larger cohort.

We recently showed that in a group of 19 patients (paired chemo-naive and NACT-treated), overexpression of insulin-like growth factor 1 receptor (IGF1R) in tumour samples (primary and metastatic) correlated with prolonged OS and disease-free survival (DFS) as compared to patients harbouring tumours with low IGF1R expression [55]. IGF1R, which is frequently overexpressed in HGSOC [56], positively correlated with high-affinity copper transporter (hCTR1) expression at both transcript and protein levels. With the further evaluation of a larger cohort, the potential of IGF1R as a prognostic marker can be established. Mitotic arrest deficiency protein 2 (MAD2), involved in the mitotic spindle assembly checkpoint, has also been found to predict response in HGSOC patients who were treated with NACT. Patients with low nuclear MAD2 staining showed poor PFS (Hazard Ratio 4.689) [57]. Similarly, checkpoint kinase 2 (CHK2) protein activation was associated with better response to platinum-based therapy in HGSOC patients [58]. High expression of prostaglandin D2 (PGD2), an inflammatory mediator produced by T-helper type 2 (T_H_2) cells and mast cells, also predicted sensitivity to platinum compounds and better DFS in patients with HGSOC [59].

#### 2.4.2. Ovarian Clear Cell Carcinomas (OCCCs)

Ovarian clear cell carcinomas comprise polygonal cells having distinct cell membranes and abundant amounts of glycogen and lipids in the cytoplasm. Primary clear cell carcinoma of the ovary accounts for <10% of EOCs and has the poorest prognosis of all EOCs. This tumour type is intrinsically resistant to chemotherapy, with only 11–27% of patients showing an objective response. They often arise from an endometriotic cyst [60]. Over the years, several protein-based biomarkers have been identified that are exclusive to clear cell carcinomas and are clinically relevant. A few of them are discussed below.

##### Diagnostic Biomarkers

Hepatocyte Nuclear Factor (HNF) 1ß: HNF 1ß is a transcription factor known to play a role in development and glucose homeostasis. Immunohistochemistry-based studies have shown that overexpression of HNF-1ß is exclusive to OCCCs as compared to other subtypes (*p* < 0.0001 each) and highlights the utility of HNF-1ß as a sensitive (sensitivity ranging from 80–100%) and specific (specificity ranging from 60–90%) biomarker for OCCC [61,62].

Napsin A: Napsin A is an aspartate protease and is a very well-known IHC marker for the diagnosis of pulmonary adenocarcinoma. It is known to promote resistance to platinum by degradation of tumour suppressor p53. In the case of EOC, several studies suggest that Napsin A is a highly sensitive (~100% sensitivity) and specific (~90% specificity) marker for the accurate diagnosis of OCCC [49,63].

AT-rich interactive domain-containing protein (ARID1A): The development of OCCC has been characterized by high mutation frequency in the *ARID1A* gene. Mutations in the *ARID1A* gene have an impact on global gene expression through dysregulated transcription and activation of major signalling pathways, which confer a survival advantage to tumour cells [64]. Loss of *ARID1A* expression is observed in approximately 50% of OCCCs, which can be readily detected by IHC staining [65]. In a study by Khalique et al., 100% concordance was observed between *ARID1A* mutational expression and immunoreactivity in EOC [66].

##### Prognostic Biomarkers

OCCCs comprise around 5% of all EOCs, with the highest incidence in the Japanese population (around 20%) [67]. Glypican-3 (GPC3), a heparan sulphate proteoglycan expressed on the cell surface in embryonic tissues, was found to be overexpressed in 44% of OCCCs, with only minimal expression in other subtypes (less than 10%). In a cohort of 213 patients, overexpression of GPC3 was associated with late-stage disease and lymph node and peritoneal metastasis as well as poor OS [68]. Thus, the expression of GPC3 in OCCC can be used as a marker to predict patients’ responses to chemotherapy. In two separate studies involving Australian and Japanese populations, the absence of nuclear staining of HNF 1ß in OCCC was associated with better OS and PFS [69]. Similarly, in a Chinese cohort, HOXA10 expression was correlated with the survival of patients, wherein the five-year survival was less than 30% in patients with positive HOXA10 expression [70]. Loss of ARID1A expression in patients who received platinum-based chemotherapy was found to be associated with poor OS [71].

#### 2.4.3. Mucinous Ovarian Carcinomas (MOCs)

Mucinous ovarian carcinomas show the presence of malignant glands and cysts that are surrounded by epithelial cells containing intracytoplasmic mucin. These tumours are often associated with endometriosis and account for about 2–3% of total EOC cases. The prevalence of this cancer is relatively uncommon and responds very poorly to the standard chemotherapeutic regimen of platinum and taxane [72].

##### Diagnostic and Prognostic Biomarker

HER-2: Human epidermal growth factor receptor 2 (HER2) is a well-known marker for breast cancer and overexpression of HER2 is associated with the aggressive behaviour of the tumour. Approximately 20% (western population) to 40% (Asian population) of mucinous ovarian adenocarcinomas show overexpression of HER2, which can be readily detected by IHC. Additionally, HER2 expression has been found to correlate with a favourable prognosis in MOC patients [73].

#### 2.4.4. Endometrioid Ovarian Carcinomas (EEOCs)

Endometroid ovarian carcinomas closely resemble the endometrium and constitute ~15% of all EOCs [40]. These tumours mostly remain confined to the ovary and are often associated with endometriosis. They are mostly negative for WT-1 and therefore can be readily distinguished from serous tumours.

##### Prognostic Biomarkers

In a cohort of 215 EEOC patients, positive expression of nuclear β-catenin, vimentin, oestrogen receptor (ER), and progesterone receptor (PR) were found to be associated with favourable outcome, while aberrant p53 expression and overexpression of *p16Ink4a* and *L1CAM* were associated with poor OS. High expression of PR in EEOC indicates a markedly favourable outcome and longer OS [74].

Despite the existence of several biomarkers (summarized in Table 2), a high mortality rate is evident due to lack of early-stage diagnosis of EOC. Therefore, there is a need to develop a robust and exhaustive panel of multiple biomarkers which potentiate early detection of the disease such that appropriate medical strategies can be adopted.

## 3. Potential Emerging Biomarkers

### 3.1. DNA-Based Biomarkers

Tumour biomarkers are routinely used in clinics to determine an accurate diagnosis. However, recent studies have also established the failure of traditional tissue-based biopsies, as they are highly invasive and provide limited sensitivity [75]. Moreover, they fail to capture the dynamic alterations in the genomic landscape of the neoplastic lesions during their gradual progression. Liquid biopsies comprising cell-free DNA (cf-DNA) and circulating tumour cells (CTCs) can better examine the molecular heterogeneity associated with disease progression, provided they are routinely carried out in patients [76]. In EOC, CTCs are identified predominantly on the basis of EpCAM expression and other epithelial markers like cytokeratin [77]. However, due to the ever-changing genetic landscape of the developing tumour, CTCs also tend to simultaneously change the expression levels of several cell surface markers. Studies have now shown that folate receptor-alpha (FRα) expression is significantly upregulated in CTCs of EOC patients and the detection of these cells increased by 92% when paired with EpCAM instead of using them alone [78]. Furthermore, during chemotherapy EOC CTCs also start expressing mesenchymal cell surface markers along with epithelial ones, which highlights the molecular heterogeneity that exists during OC development [79]. cf-DNA is often present at high concentrations in the blood of EOC patients and can be used to detect molecular changes such as microsatellite instability (MSI) and loss of heterozygosity (LOH) as well as epigenetic changes such as DNA methylation in the cancer cells [80]. Studies have successfully established the utility of both cf-DNA and CTCs in predicting the response to therapy as well as identification of mutations which could be used for diagnosis as well as prognosis [81]. Additionally, epigenetic modifications such as methylation occur very early in cases of malignant transformation and can be detected in cf-DNA up to two years before diagnosis, thereby aiding the identification of prospective candidates for personalized therapy [82,83]. This warrants the need to constantly enhance our knowledge of the dynamic genomic and proteomic landscape and simultaneously include these findings in an exhaustive panel of markers that can be used for EOC detection.

### 3.2. RNA-Based Biomarkers

Novel RNA biomarkers are emerging as useful tools for both diagnosis and elucidating the prognosis of ovarian cancer. As a diagnostic tool, RNA has the advantage of easy detection and quantification even at very low concentrations. Owing to its multiple copies within the cell, it provides dynamic insights into the cellular status and are easily detected at the early stages of disease progression [84]. High-throughput sequencing has allowed the identification of a plethora of novel coding (mRNA) and non-coding RNAs (small nucleolar RNA, small nuclear RNA, microRNA, etc.) that are differentially expressed in different forms of cancer. Circular RNA (circRNA) is one such novel RNA subtype, with a length ranging from a hundred to several thousand nucleotides. Ning et al. were successful in identifying a differential pattern in circRNA expression in EOC [85]. circN4BP2L2, a splice variant of N4BP2L2 mRNA, was found to be significantly downregulated in EOC. Their results not only established the diagnostic power of circN4BP2L but also indicated its prognostic value. Similar studies conducted on several novel circRNAs such as circBNC2 and circSETDB1 have shown incredible potential as biomarkers in the diagnosis, classification, and therapy response to EOC [86,87]. LncRNAs have been shown to play a crucial role in tumorigenesis. Lv et al., upon analysis of EOC-related GEO datasets, observed a significant downregulation in the expression of a novel tumour-suppressing lncRNA, small nucleolar RNA host gene 10 (SNHG10). They further reported SNHG10 to act as an miRNA sponge and observed its upregulation to be negatively correlated with poor prognosis [88]. Analysis of transcriptomic data by Zalfa et al. has further yielded a set of eight genes that can discriminate EOC patients from healthy ones with 100% specificity and sensitivity [89]. Furthermore, Saral et al. have been successful in the identification of a set of three miRNAs (miR-16-5p, miR-17-5p, and miR-638) that proved to be potential non-invasive biomarkers in the identification of EOC patients [90]. Clinical trials are also being conducted to identify the expression of EOC-specific RNAs with both diagnostic and prognostic use (NCT03738319, NCT02785731, NCT05030805).

### 3.3. Exosome-Based Biomarkers

The markers that have been mentioned until now are directly obtained either from serum or plasma. However, the environment surrounding these biomolecules can be hostile, leading to their degradation and compromising their diagnostic power. Therefore, use of exosome-coated biomolecules is a significant improvement in sensitivity and specificity due to their enhanced stability [91]. Exosomes are lipid-coated vesicles that originate from constant blebbing of the endocytic reticulum. These are routinely secreted into the extracellular region and are considered crucial to cellular signalling [92]. Exosome-based liquid biopsy is rapidly gaining precedence over traditional biopsies, predominantly due to a significant improvement in their stability in blood and ease of isolation from other subcellular vesicles. Most importantly, they encapsulate a variety of different biomolecules such as proteins, lipids, DNA, and RNA as well as miRNA. Taylor and Taylor carried out profiling of miRNA from the exosomes and the tumour bulk. They reported that exosomal miRNA profiling can be used as a surrogate tool for carrying out tissue miRNA expression analysis for future diagnostic purposes [93]. The advantages of using exosome-based biomarkers in cancer diagnosis over the existing ones have been reviewed in depth by Yu et al. [94]. A plethora of researchers are currently investigating the clinical relevance of exosomal miRNA and proteins [95,96,97]. Recently, Jeon et al. reported miR-1290 to be a promising discriminator between HGSOC versus benign ovarian neoplasm. They performed receiver operating curve (ROC) analysis and used the area under curve (AUC) to assess the diagnostic potential of miR-1290; they observed that upon pairing it with CA125, the AUC value increased significantly from 0.812 to 0.954, indicating its potential as a future clinical biomarker [98]. Exosomal miRNA-205 has previously been reported to be instrumental in tumorigenesis by being multi-functional in cellular processes such as cellular proliferation in melanoma cells [99], angiogenesis in thyroid cancer [100], EMT [101], and chemoresistance in breast cancer [102]. A pioneering study conducted by Zhu et al. evaluated the diagnostic power of exosomal miR-205 in EOC patients. They observed that upon combining miR-205 singly with the traditional markers CA125 and HE4, the AUC increased from 0.915 to 0.930 and from 0.779 to 0.827, respectively. Furthermore, the combination of these three markers together increased the AUC to 0.951, sensitivity to 100%, and specificity to 86.1% [67]. Along with miRNAs, exosomes also tend to accommodate proteins as their cargo, which significantly enhances their stability in the serum. Zhang et al. conducted the proteomic profiling of exosomes isolated from the patient’s plasma, which yielded four differentially expressed proteins. Amongst them, the fibrinogen alpha chain (FGA) was found to be consistently upregulated in the proteomic analysis and the cancer cohort of the patient sample analysis. It also indicated FGA to have a higher diagnostic potential, with an AUC of 0.8459 [103]. Multiple clinical trials are also underway to identify the exosomal miRNA signature in OC (both epithelial and endometroid) versus benign cases and study their diagnostic potential (NCT03738319, NCT03776630, NCT03738319) and indicators of treatment response (NCT01391351). Therefore, exosome-encapsulated biomolecules provide a non-invasive and effortless tool toward EOC diagnosis.

### 3.4. Protein-Based Biomarkers

Osteopontin is a glycoprotein present in the extracellular matrix and is secreted by osteoblasts and endothelial cells. It is one of the early tumour biomarkers in the blood known to be elevated in EOCs. However, no significant difference in its expression is observed across various subtypes of EOCs, thereby limiting its utility as a subtype-specific biomarker. The sensitivity and specificity of osteopontin are approximately 90% in serum and 98% in ascites in HGSOC patients [104]. In a study by Lan et al., osteopontin combined with CA-125 presented a higher AUC than osteopontin alone (0.93 vs. 0.91) [105]. Moreover, CA-125 in combination with other biomarkers such as cVCAM, CA 19-9, EGFR, and eotaxin increased the sensitivity and specificity up to 98% for the early detection of EOC. Additionally, 50% of EOC patients show overexpression of interleukins, particularly IL-6 and IL-8. Therefore, these proteins can be readily used as early-stage biomarkers for EOC [106].

Several groups have now designed novel biomarker panels comprising conventional clinical markers such as CA-125 and HE4 and novel candidate biomarkers such as MK, hK11, KLK6, FRα, PRSS8, and IL-6 for early detection of EOC among the general female population using ProSeek technology [107]. Recently, glycan-based biomarkers have been developed that take advantage of the fact that multiple unusual modifications of glycans have been reported in different forms of cancer. However, the majority of the findings in glycomic profiling of tumours are in the discovery phase and have not received clinical approvals yet [108].

### 3.5. Copper Isotopes

Copper is a trace element deemed essential for organisms. Metabolic changes in copper levels have been reported to cause a proangiogenic effect during carcinogenesis. More recently, the ^63^Cu/^65^Cu isotopic ratio (δ^65^Cu) has been observed to be modified in the serum of breast and colorectal cancer patients. A higher level of the lighter isotope was observed in the serum, whereas the levels of the heavy isotope were high in the tumour mass [109]. This variation in copper levels can be attributed to increased glycolysis and high lactate production in the cancerous cells. δ^65^Cu is gradually emerging as a novel diagnostic marker owing to its greater turnover time (~6 weeks) during carcinogenesis in comparison to other protein markers in different cancers [110]. A study conducted by Toubhans et al. revealed that serum δ^65^Cu levels were significantly lower in 44 EOC patients when compared to healthy volunteers [111]. This result is in tandem with the results obtained by Telouk et al., which further seems to strengthen the potential of using δ^65^Cu as a diagnostic tool in the future [109].

## 4. Emergence of Bioinformatics

With the sheer amount of data being generated, better technologies must be created to curate and store this data for retrospective analysis. Even though necessary steps are being taken toward mining this “big data”, steps fall short, especially in oncology [112]. The National Cancer Institute (NCI) has been at the forefront of creating advancements in cancer bioinformatics. The NCI Centre for Biomedical Informatics and Information Technology (CBIIT) has been developed to oversee such bioinformatic-related initiatives. Multiple such databases and repositories such as the National Cancer Informatics Program (NCIP), Gene Omnibus Database (GEO), etc. currently exist, which act as points of liaison to integrate both research-based and patient-derived data [113]. Similarly, multiple groups are utilizing such bioinformatic tools to predict the diagnostic/prognostic potential of several differentially regulated genes (DEGs). For a better overview of these DEGs in a specific cancer type, the datasets obtained from microarray technology are being utilized [114]. This allows the analysis of the high-throughput and swift generation of data that is heavily reliable and has translational potential. Liu et al. successfully utilized five datasets that were obtained from the GEO, the Cancer Genome Atlas (TCGA), and Genotype-Tissue Expression (GTEx). They observed 590 DEGs, which included 276 genes that were upregulated and 314 that were downregulated. A plethora of exhaustive in-silico studies yielded nine different genes showing putative diagnostic potential using two different machine-learning algorithms [115]. In-silico approaches have also been adapted to identify the prognostic value of multiple genes. Cao et al. carried out a meta-analysis of four different datasets in the Oncomine database to observe an upregulation in TET3 mRNA levels in ovarian cancer. They also observed an upregulation of TET3 expression in advanced-stage serous ovarian cancer patients. Interestingly, they found TET3 to be associated with both poor OS and PFS [116]. A deluge of such studies currently exist that have successfully been able to identify proteins that can be used as promising biomarkers in the future [117,118,119,120]. However, it is important to inculcate a multidisciplinary approach in studying the diagnostic/prognostic value of any molecular marker. Using bioinformatics singly is not sufficient to establish any merit of the diagnostic/prognostic prowess of a marker. It requires an acute involvement of in-silico, in-vitro, and in-vivo studies to better complement and corroborate the findings obtained at every stage of the study. This allows better identification of molecular markers and also leads to the development of better treatment strategies [121].

## 5. Conclusions

The past decade has witnessed a boom in the development of high-throughput technologies, especially in the field of genomics and proteomics. This has led to the identification of several potential biomarkers and has also provided new insights into the diagnosis/prognosis of EOC. However, only a few with promising diagnostic/prognostic value have been able to cross over into clinical settings. More exhaustive research in this particular field has led us to the conclusion that a single biomarker does not have the prowess to diagnose EOC effectively in every single patient. This warrants a panel of new biomarkers that allow the early-stage detection of EOC. The approaches and/or technologies mentioned in this review hold significant value in discovering more robust biomarkers for diagnosis, prognosis, or prediction of therapy response in EOC. Research on ovarian cancer presently focuses on three main aspects: first is the necessity of rigorous validation such that more and more putative biomarkers can be successful in clinical trials. Second is the constant need to discover and upgrade our knowledge of more specific and sensitive novel biomarkers. Lastly, multiple studies posit the necessity of generating a panel of biomarkers such that early-stage detection can be achieved. Emerging technologies, in-silico approaches, and free sharing of data amongst the scientific community can help in furthering these goals.

## Figures and Tables

**Table 1:** p53/p16 index.

P53/p16 Index	High-Grade Serous	Low-Grade Serous
P53	>70% of cells (independent of p16)	>1% and <70% cells
P16INK4A	>90% of cells in case of complete absence of p53	<90% of cells

**Table 2:** List of current IHC-based biomarkers used in ovarian cancer diagnosis.

Biomarker	Clinical Utility	Subtype	Clinical Role
Wilms Tumour 1 (WT-1)	Diagnosis/Prognosis	High-Grade Serous	Diagnosis—Allows distinguishing HGSOC from other subtypes Prognosis—Co-expression of WT-1 with ER-alpha indicates a good prognosis
P53	Diagnosis	High-Grade Serous	Strong nuclear expression or total loss of expression in IHC
P16	Diagnosis	High-Grade Serous	Diffused staining is observed in IHC
Ki-67	Prognosis	Low-Grade Serous	High expression is negatively correlated to OS
Estrogen Receptor (ERα)	Prognosis	Low-Grade Serous	High expression is associated with better survival outcomes
IGF1R	Prognosis	High-Grade Serous	Higher expression is positively correlated with OS and DFS
Mitotic Deficiency Protein (MAD2)	Prognosis	High-Grade Serous	Low nuclear staining is associated with poor PFS
Checkpoint Kinase 2 (CHK2)	Prognosis	High-Grade Serous	High CHK2 expression is associated with a better response to chemotherapy
Prostaglandin 2 (PGD2)	Prognosis	High-Grade Serous	High expression is positively correlated to therapy response and DFS
Hepatocyte Nuclear Factor 1 β (HNF-1β)	Diagnosis	Ovarian Clear Cell Carcinoma	>75% of cells show strong nuclear staining
Napsin A	Diagnosis	Ovarian Clear Cell Carcinoma	Moderate or coarse cytoplasmic granular staining
ARID1A	Diagnosis	Ovarian Clear Cell Carcinoma	Fair or strong nuclear staining
Glypican–3 (GPC3)	Prognosis	Ovarian Clear Cell Carcinoma	High GPPC3 expression is associated with late-stage metastasis
Homeobox A10 (HOXA10)	Prognosis	Ovarian Clear Cell Carcinoma	Higher expression is negatively correlated to 5-year survival
Human Epidermal Growth Factor Receptor 2 (HER2)	Diagnosis/Prognosis	Mucinous Ovarian Carcinoma	Diagnosis—Strong membranous staining Prognosis—High expression is positively correlated with a favourable prognosis
Progesterone Receptor (PR)	Prognosis	Endometroid Ovarian Carcinoma	Higher expression is associated with a longer OS

## Data Availability

No new data were created or analyzed in this study. Data sharing is not applicable to this article.
